# Cross-cultural translation and measurement properties of the Polish version of the Knee injury and Osteoarthritis Outcome Score (KOOS) following anterior cruciate ligament reconstruction

**DOI:** 10.1186/1477-7525-11-107

**Published:** 2013-06-27

**Authors:** Przemysław T Paradowski, Dariusz Witoński, Rafał Kęska, Ewa M Roos

**Affiliations:** 1 Department of Reconstructive Surgery and Arthroscopy of the Knee Joint, Faculty of Health Sciences, Medical University, Radliński Hospital, Drewnowska 75, 91-002 Łódź, Poland; 2 Department of Orthopedics, Sunderby Central Hospital of Norrbotten, SE-971 80 Luleå, Sweden; 3 Department of Orthopedics, Haugesund Hospital, Helse Fonna, Karmsundgata 120, N-5528 Haugesund, Norway; 4 Research Unit for Musculoskeletal Function and Physiotherapy, Institute of Sports Science and Clinical Biomechanics, University of Southern Denmark, Campusvej 55, DK-5230 Odense M, Denmark

**Keywords:** Knee injury and Osteoarthritis Outcome Score, Patient-reported outcome, Validation study, Psychometrics, Anterior cruciate ligament, Orthopedic surgery

## Abstract

**Background:**

Knee Injury and Osteoarthritis Outcome Score (KOOS) is available in over 30 languages and a commonly used Patient-Reported Outcome (PRO) for assessment of treatment effects following knee surgery. The aim of the study was to report the linguistic translational process and evaluate the psychometric properties of the Polish version of the KOOS questionnaire.

**Methods:**

We translated and culturally adapted the KOOS according to current guidelines for use in Poland. Patients who had undergone anterior cruciate ligament reconstruction (ACLR) completed the KOOS and Short Form 36 Health Survey (SF-36). We evaluated floor/ceiling effects, reliability (using Cronbach’s alpha, intraclass correlation coefficients (ICC) and measurement error), convergent and divergent construct validity (using four *a priori* stated hypotheses) and responsiveness (using data obtained prior to and one year after ACLR and described by both effect size (ES) and standardized response mean (SRM)).

**Results:**

The clinical study population consisted of 72 subjects (mean age 29.8, 28% women). We did not observe floor effects in any KOOS subscales neither pre- nor postoperatively. As expected, ceiling effects were found postoperatively for the subscales Pain and ADL in this cohort assessed on average 1.3 year after surgery as more than 15% reported no pain or limitations in daily activities. The Cronbach’s alpha was above 0.9 for all subscales indicating excellent internal consistency. The test-retest reliability of all KOOS subscales at one-year postoperatively was excellent with ICCs exceeding 0.86 for all subscales. The minimal detectable change on group level ranged from 1.3 to 2.4, and on an individual level from 10.9 to 20.2. Responsiveness was demonstrated since the expected pattern of effect sizes between subscales following ACLR was found.

**Conclusions:**

We found the Polish version of the KOOS to be a valid and reliable instrument for use in patient groups having ACLR. We caution against monitoring individual patients since the smallest change considered clinically relevant cannot reliably be detected.

## Background

Functional disability and quality of life are the key outcomes that influence patients’ compliance and satisfaction with treatment [1]. It has already been well-established that both functional status and quality of life can be better described by patients themselves than on the basis of clinical examination made by a physician [2,3]. Patient-reported outcomes (PROs) are thus necessary for clinical research purposes across countries and cultures. The measurement instrument should be standardized, sensitive to clinical change, concise, and convenient for both the patients evaluated and clinicians [4]. To enable data collection from patients speaking different languages and living in different cultures, questionnaires should undergo linguistic and cross-cultural translation processes and psychometric properties should be assessed in clinical validation studies [5].

The Knee injury and Osteoarthritis Outcome Score (KOOS) [6,7] is such a commonly used PRO, originally developed simultaneously in English and Swedish and currently available in 39 different languages [8]. The KOOS has already been validated for use in the United States [6], Sweden [7], Singapore [9], Iran [10], France [11], the Netherlands [12], Portugal [13], and Japan [14]. KOOS was developed to assess difficulties experienced by patients with joint injuries and knee OA and is commonly used to evaluate the effect of orthopedic surgery including Anterior Cruciate Ligament Reconstruction (ACLR) [6,7].

Until now there were no internationally established and formally cross-culturally adapted PROs that could be used for assessment of functional status and quality of life following knee surgery in Poland.

The aim of this study was therefore to first linguistically and cross-culturally translate and second to test the psychometric properties of the Polish version of the KOOS questionnaire as expressed by reliability, validity and responsiveness, in a cohort of patients having had ACLR.

## Methods

### Linguistic and cross-cultural translation process

The process followed the recommendations by Beaton et al. [5]. The KOOS was simultaneously developed in English and Swedish (two original languages). A total of five persons were involved in the translational process. Two independent forward translations (T1, T2) were performed from the English version by an orthopaedic surgeon, fluent in English with Polish origin, and a professional language translator. One independent translation (T3) was performed from the Swedish version by a medical professional fluent in Swedish with Polish origin. The three versions were then unified in a consensus meeting. Two native English-speaking persons of Polish origin (BT1 and BT2), with medical and technical professions respectively, backwards translated independently this new version into English. Both translators were unfamiliar with the original questionnaire and its concept. During the expert meeting in which all translators participated all versions of the KOOS questionnaires were combined and consensus on semantic, idiomatic, experiential and conceptual equivalence was reached resulting in a pre-final version of the questionnaire. This version was pre-tested on 10 patients with ACL injury (six men and four women with a mean and median age of 34 years, range 20–54) prior to ACLR. The patients were asked whether they fully understood the items, if they found any items ambiguous and whether they had any problems in answering the items. The Polish version of the KOOS is available free of charge from http://www.koos.nu[8].

### Clinical validation study

#### Patients

All patients were native Polish speakers with an intermediate or higher educational level. They were operated and followed up at the Department of Reconstructive Surgery and Arthroscopy of the Knee Joint, Medical University in Łódź between January 2007 and August 2009. The subjects had undergone reconstructive ACL surgery and, in case of combined injury both ACL reconstruction and meniscal resection. The reconstruction was made endoscopically with bone–patellar tendon–bone grafts that were stabilized with titanium interference screws. All patients had undergone standardized moderately accelerated, six months’ rehabilitation program [15]. The mean follow-up time was 1.3 years (0.4–3.4). At time of follow-up all subjects had returned to their normal activities.

Participants were asked to complete the Polish version of KOOS three times: first preoperatively, then during the 1-year routine follow-up and finally for test-retest purposes one till two weeks following the 1-year routine follow-up. Patients filled out the KOOS in the clinic the first two times and at home the third time. Questionnaires were returned by ordinary mail. A one to two weeks test-retest time interval is considered appropriate and previously used for the KOOS [6,7,16]. The SF-36 [17] (licence number H1 031207-30347) was administered once at the 1-year postoperative follow-up. The study was approved by the ethics committee at the Medical University of Łódź. An informed written consent was obtained from all subjects participating in the study.

#### Questionnaires

The KOOS is a 42-item self-administered knee-specific questionnaire assessing pain (9 items), symptoms (7 items), activities of daily living (ADL, 17 items), sports and recreation function (5 items) and knee-related quality of life (QOL, 4 items) in five separate subscales. Each item is responded to by marking one of five response options on a Likert scale. A score from 0 (extreme problems) to 100 (no problems at all) is calculated separately for each subscale.

The Short Form 36 (SF-36) Health Survey includes 36 items that are combined in eight subscales: physical functioning (PF), role-physical (RP), bodily pain (BP), general health (GH), vitality (VT), social functioning (SF), role-emotional (RE) and mental health (MH), and one single-item measure of health transition which is not used in scoring the scales or summary measures. A score from 0 (worst possible health status) to 100 (best possible health status) is independently generated for each subscale. The SF-36 had already been validated in Polish [18].

#### Statistical analysis

Analyses were performed with use of SPSS for Windows 15.0.0 (SPSS, Chicago, IL, USA). We considered a two-tailed *P* less than 0.05 to be significant.

#### Missing items

In accordance with the 2003 Users Guide, for the KOOS questionnaire two missing items were allowed in each subscale. Missing data were then subsequently imputed with the mean of other values within the same subscale [8]. SF-36 results were calculated using standard scoring procedures whereby missing values were replaced by scale means where valid responses were available for at least half of of the scale items [17].

#### Floor/ceiling effects

Floor or ceiling effects were assessed pre- and postoperatively and considered to be present if more than 15% of the respondents achieved the lowest or highest possible scores [19]. Floor and ceiling effect may differ due to when (pre- or postoperatively) the questionnaire is administered. Preoperatively floor effects can be expected since experiencing symptoms is an indication for surgery. Post-operatively, if anything, ceiling effects can be expected if the intervention has been successful and the patient has returned to all normal activities and have no symptoms.

### Reliability

#### Internal consistency

Internal consistency is defined as the degree of the interrelatedness among the items. It was determined by calculating Cronbach’s alpha coefficient. Cronbach’s alpha was determined at the first 1 year follow-up assessment. Cronbach’s alpha value of more than 0.70 was considered as satisfactory [20].

#### Test-retest reliability

Reliability is a proportion of the total variance in the measurements due to differences between patients. Test-retest reliability of the KOOS subscales was assessed 1 year postoperatively using the two administrations completed at a one to two week interval. It was assumed that the probability of a spontaneous significant change in clinical status was low during a one to two week interval. The test-retest reliability of the KOOS was analyzed using two-way random effect model of the intra-class correlation coefficient (ICC) for absolute agreement and presented with 95% confidence interval (CI). An ICC equal to or greater than 0.80 was considered acceptable for groups and an ICC of more than 0.90 for individual patient use [20-24].

#### Measurement error

Measurement error is the systematic and random error of a patient’s score that is not attributed to true change in the construct to be measured. Standard error of measurement (SEM) for absolute agreement was calculated based on the standard deviation (SD) of the sample and the reliability of the measurement instrument according to the following formula: SEM = SD √(1-R) where R represents the reliability parameter (ICC) [23]. Then, in turn, the minimal detectable change (MDC), which is the threshold for determining clinical changes outside measurement error, was calculated on the basis of the SEM of the test–retest reliability using the following formula: MDC = SEM × 1.96 × √2, where 1.96 derives from the 0.95% confidence interval of no change and √2 represents two measurements evaluating the change [23,24]. The MDC can be modified for group comparison (for research purposes), depending on the size of the group (*n* = 72), as follows: MDCgroup = MDCindividual/√n [24].

The MDC should preferably be smaller than the Minimal Important Change (MIC). MIC is the smallest change score needed for the effect to be considered clinically relevant [25]. Overall, a MIC of 8-10 points was considered to be appropriate for the different KOOS subscales [26]. However, it must be acknowledged that the MIC is dependent on context factors, including patient group, intervention and time to follow-up. Therefore, it is more appropriate to establish the MIC for specific contexts. The MIC for KOOS at 1 year following ACLR has not yet been determined.

### Validity

#### Construct validity

Construct validity is defined as the degree to which the subscales of the KOOS measures the characteristic to be measured. Convergent and divergent construct validity was determined by comparing the results of the subscales of KOOS and SF–36 questionnaires representing similar and dissimilar constructs. The Spearman’s rank correlation was used to assess the association between domains. Correlation coefficients greater than 0.5 were considered strong, correlations between 0.35 and 0.5 moderate and less than 0.35 were considered weak [27]. A priori hypotheses had been generated to determine convergent (when moderate or strong correlation is expected) and divergent (weak correlation expected) construct validity. It was hypothesized that 1) KOOS ADL should correlate with at least 0.35 with SF–36 PF, 2) KOOS Sports and Recreation Function should correlate with at least 0.35 with SF–36 PF, 3) KOOS Pain should correlate with at least 0.35 with SF–36 BP, and finally that 4) all KOOS subscales should correlate stronger with SF–36 subscales representing Physical Health (PF, RP, BP) than with SF-36 subscales representing Mental Health (GH, VT, SF, RE, MH).

### Responsiveness

Responsiveness is the ability to detect changes of the construct of interest over time. Since Global Perceived Effect (GPE) was not assessed postoperatively this approach was not possible. We thus chose to calculate effect size (ES) defined as score change divided by baseline SD [28] and set up a priori hypotheses regarding the pattern of effect sizes between subscales. Based on the pattern seen at 1 year in the Danish ACLR-registry [29] it was hypothesized that the subscale QOL would show the largest ES (over 1.0), followed by the subscale Sports and Recreation Function. The subscales Pain, Symptoms and ADL would have lower ES, at least 0.15 below Sport and Recreation Function. By focusing on the expected pattern and not on the absolute values of ES the focus is on the measurement properties of the PRO studied and not on the clinical effect seen from the intervention which is dependent on factors such as patient selection and concomitant injuries. In addition to ES, responsiveness was also presented as standardized response mean (SRM). SRM was calculated by dividing the mean score change with the standard deviation of that score change [30]. The same pattern was expected for SRM:s and the five KOOS subscales.

To compare KOOS scores before ACLR and at follow-up the Wilcoxon signed rank test was used.

## Results

### Linguistic and cross-cultural translation process

The Polish version of the pre-final KOOS questionnaire was well-accepted in the pre-test. All questions and response options were considered satisfactory and understandable by the subjects. However, in order to improve clarity minor changes were made for two items according to patients’ suggestions. In item A13 the expression “wchodzenie/wychodzenie z wanny/spod prysznica” was considered better corresponding to English phrase “getting in/out of bed” than the previously used phrase “korzystanie z wanny/natrysku”. In item A15 we replace the phrase “korzystanie z toalety” with “siadanie na sedesie/wstawanie z sedesu” that more precisely described “getting on/off toilet”. The revised version of the questionnaire was again assessed as semantically, idiomatically and conceptually equivalent with the original version. The revised version was used in the clinical validation study.

### Clinical validation study

#### Patients

In total, 72 subjects were enrolled in the study. Of them 48 (67%) had undergone ACLR alone and 24 (33%) ACLR and concomitant meniscal resection. Patient characteristics are given in Table 1.

**Table 1:** Characteristics of included subjects having anterior cruciate ligament reconstruction (ACLR)

**Characteristics**	**ACLR**
*N* (% women)	72 (28)
Age at surgery, mean (SD) years	29.8 (9.2)
Time to follow-up after surgery, mean (SD) years	1.3 (0.6)
Tegner score at follow up, median (range)	4 (3–8)

#### Missing items

A subscale score could be calculated for all KOOS subscales at both administrations. At baseline, a total of eight items out of the possible 42 (number of items) × 72 (number of patients) or 0.26% were missing. At follow-up, six KOOS items (0.2%) were missing. For the SF-36 the number of missing items at follow-up were 12 (0.46%).

#### Floor/ceiling effects

Preoperatively, there were neither floor nor ceiling effects (determined as >15% having worst or best possible score) in any of the KOOS subscales. Best possible scores were reported by 13%, 11%, 3% and 3% for the subscales ADL, Pain, Symptoms and Sports and Recreation Function, respectively. No subjects reported best possible score in subscale QOL prior to surgery.

At the 1-year follow-up there were no floor effects (indicating worst possible status) in any KOOS subscales. As expected, ceiling effects were found after surgery for the subscales Pain (19%) and ADL (29%). Best possible scores were reported by 13%, 11%, and 3% for the subscales Symptoms, Sports and Recreation Function and QOL, respectively.

### Reliability

Median number of days from test to retest was 7 (range 5-19).

#### Internal consistency

Cronbach’s alpha ranged from 0.92 to 0.97 indicating excellent internal consistency (>0.8) of all subscales both pre- and postoperatively, Table 2.

**Table 2:** Mean KOOS scores (0 to 100, worst to best scale) at test and retest administrations one week apart, test-retest reliability, internal consistency and minimal detectable change of KOOS subscales for individuals and groups 1.3 year after anterior cruciate ligament reconstruction (ACLR)

**KOOS subscales (number of items)**	**Mean KOOS score (SD)**		**ICC (95% CI)**	**Cronbach’s alpha coefficients**	**SEM**	**Minimal detectable change (95% CI) in individuals**	**Minimal detectable change (95% CI) in groups**
	**First follow-up assessment**	**Second follow-up assessment**					
ACLR, n = 72							
Pain (9)	88.1 (13.4)	87. 7 (13.9)	0.86 (0.84–0.88)	0.92	5.1 (4.6–5.4)	14.1 (12.9–14.9)	1.7 (1.6–1.8)
Symptoms (7)	81.9 (16.9)	82.2 (16.4)	0.90 (0.89–0.92)	0.95	5.2 (4.8–5.6)	14.5 (13.3–15.6)	1.7 (1.5–1.8)
ADL (17)	92.1 (12.1)	92.1 (11.9)	0.89 (0.88–0.91)	0.94	3.9 (3.6–4.2)	10.9 (10.1–11.6)	1.3 (1.2–1.4)
Sports/Rec (5)	72.0 (24. 9)	71.5 (26.2)	0.93 (0.91–0.94)	0.96	6.8 (6.1–7.5)	18.8 (16.9–20.7)	2.2 (2.0–2.4)
QOL (4)	63.8 (23.1)	65.4 (22.4)	0.90 (0.88–0.92)	0.95	7.3 (6.5–8.0)	20.2 (18.1–22.2)	2.4 (2.1–2.6)

Abbreviations: *ICC* Intraclass correlation coefficient, *SEM* Standard error of measurement, *ADL* Activities of daily living, *QOL* Quality of life.

#### Test-retest reliability

The reliability of all KOOS subscales was excellent (>0.8) with ICCs ranging from 0.86 to 0.93 (Table 2).

#### Minimal detectable change

At group level, MDC ranged from 1.7 to 2.4. At the individual level the MDC was lowest (10.9) for KOOS ADL and highest (19.9) for KOOS QOL (Table 2).

### Validity

#### Construct validity

All a priori established hypotheses were supported. We confirmed high correlation between KOOS Pain and SF–36 BP (r_s_ = 0.66), KOOS ADL and SF–36 PF (r_s_ = 0.65) and between KOOS Sports and Recreation Function and SF–36 PF (r_s_ = 0.67). All KOOS subscales correlated stronger with SF–36 subscales of Physical Health (PF, RP, BP) than with subscales representing Mental Health (GH, VT, SF, RE, MH) except for the correlation between KOOS subscale Symptoms and SF–36 RP (Table 3).

**Table 3:** Construct validity, given as Spearman correlations of the five KOOS subscales and the eight SF-36 subscales in subjects following anterior cruciate ligament reconstruction (ACLR) (n = 72)

		**KOOS subscales**				
		**Pain**	**Symptoms**	**ADL**	**Sport/Rec**	**QOL**
SF-36 subscales	PF	0.68	0.45	0.65*	0.67*	0.64
	RP	0.56	0.29	0.47	0.50	0.43
	BP	0.66*	0.41	0.54	0.51	0.51
	**GH**	**0.38**	**0.18**	**0.38**	**0.33**	**0.39**
	**VT**	**0.38**	**0.21**	**0.35**	**0.32**	**0.36**
	**SF**	**0.38**	**0.23**	**0.38**	**0.33**	**0.25**
	**RE**	**0.22**	**0.12**	**0.20**	**0.25**	**0.17**
	**MH**	**0.17**	**0.08**	**0.12**	**0.10**	**0.14**

Abbreviations: *ADL* Activities of daily living, *QOL* Quality of life, *PF* Physical functioning, *RP* Role-physical, *BP* Bodily pain, *GH* General health, *VT* Vitality, *SF* Social functioning, *RE* Role-emotional, *MH* Mental health.

* As hypothesized, expected correlations were above 0.35 for *a priori* hypotheses 1-3.

As hypothesized, correlations between KOOS subscales and SF-36 subscales better representing Mental Health (bolded in table) were lower than between KOOS subscales and SF-36 subscales better representing Physical Health (*a priori* hypothesis 4).

### Responsiveness

Effect sizes ranged from 0.41 to 1.38 and standardized response means ranged from 0.39 to 1.08 for the five KOOS subscales. Our a priori hypotheses were confirmed in that the largest ES was seen for the subscale QOL (1.38) followed by Sports and Recreation Function and thereafter with a difference of at least 0.15 the subscales ADL, Symptoms and Pain (Table 4).

**Table 4:** Mean KOOS scores (0 to 100, worst to best scale), before anterior cruciate ligament reconstruction (ACLR) and at one-year follow-up (N = 72)

**KOOS subscales**	**Before ACLR mean (SD)**	**1yr follow-up mean (SD)**	***P***	**Effect size**	**Standardized response mean**
Pain	80.6 (16.0)	88.1 (13.4)	<0.001	0.41	0.39
Symptoms	74.6 (18.0)	81.9 (16.9)	0.002	0.47	0.49
ADL	84.0 (15.3)	92.1 (12.1)	<0.001	0.53	0.58
Sports/Rec	48.4 (26.9)	72.0 (24.9)	<0.001	0.88	0.80
QOL	39.0 (18.0)	63.8 (23.1)	<0.001	1.38	1.08

Responsiveness is given as effect sizes and standardized response means for all KOOS subscales.

*ADL* Activities of daily living, *QOL* Quality of life. Responsiveness is given as effect size and standardized response mean. Wilcoxon signed rank test was used for comparison of KOOS subscales’ scores before ACLR and at follow-up. KOOS is 0-100 points, worst to best.

## Discussion

The study reports on the linguistic and cross-cultural translation and the psychometric properties of the Polish version of the KOOS in patients having had ACL reconstruction. To improve the quality of the report, cross-cultural translation of the KOOS for use in the Polish language was reported in accordance with the COSMIN checklist for cross-cultural validation [31,32]. The COSMIN checklist was easy to work with and can be recommended for future reports of cross-cultural translation and validation processes [33].

The results indicate that the Polish version of KOOS questionnaire is a reliable, valid and responsive tool for use in groups of patients having ACL reconstruction.

In this study Cronbach’s alphas ranged from 0.92 to 0.97 indicating very high internal consistency. These values are higher than in previous KOOS validation studies [6,9,11-13]. One possible explanation is the relative homogeneity of the group examined. We evaluated reliability at one year postoperatively when patients likely constitute a more homogenous sample compared to pre-operatively, a time point frequently used by others for assessment of reliability.

We found the test-retest reliability to be excellent with ICCs ranging from 0.86 to 0.93. It revealed satisfactory stability and reliability of all the KOOS subscales over time in examined subjects. The ICCs observed in our study were higher than in previous studies in patients with knee injuries [6,7] and osteoarthritis [9,13]. Explanations include test-retest reliability being assessed postoperatively in this sample while others commonly use preoperative samples. ICCs comparable to ours were observed by de Groot et al. in validation of the Dutch version of the KOOS in patients with different stages of osteoarthritis (OA) [12]. They found that the highest ICCs occurred in subjects with moderate OA. Since the patients with mild OA as well as those after revision total knee replacement had lower ICCs, especially in the KOOS subscale Sports and Recreation Function, they suggested that the questions about sport were less relevant in these groups. Such a phenomenon was not observed in our study.

The excellent test-retest reliability translated into smallest detectable changes of 3 points or less for the different subscales. Being able to detect a difference of 3 points indicates that the currently suggested minimal clinically important change of KOOS of 8-10 [26] is well detectable in groups examined. However on an individual level, greater changes are needed (10.9 to 20.2) for the different subscales to be reliably detected. This means that despite excellent reliability the KOOS is better used for monitoring groups of subjects.

We confirmed content validity at the pre-test evaluation in that the original questionnaire items were relevant for young active individuals undergoing ACLR in Poland. Considering the large number of KOOS translations available in countries with a similar cultural context, we did not ask patients to add items to the existing questionnaire. The construct validity of the KOOS questionnaire was determined by setting up a priori hypotheses and comparing the KOOS subscales with the subscales of the SF–36. We compared the correlations between respective subscales measuring similar or dissimilar constructs. As hypothesized, the highest correlations were observed between SF–36 subscales and KOOS subscales measuring similar constructs while low correlations were seen when comparing subscales measuring dissimilar constructs. Correlation coefficients were comparable to those previously seen by Roos et al. [7] and Goncalves et al. [13].

The results of the responsiveness assessment confirmed that KOOS is able to detect clinical improvement in subjects undergoing ACLR. Further we confirmed the a priori set hypotheses including QOL being the most responsive subscale following ACLR. The pattern found was similar to data reported by Lind et al. in a 2-year follow-up study of 5000 patients having had primary ACLR included in the Danish knee ligament reconstruction registry [30].

There are limitations to be acknowledged. Since psychometric properties of a questionnaire may depend on the characteristics of the patients included, our findings apply to young adults having ACLR only and not necessarily to elderly with OA or those having other interventions. Further validation of the Polish version of the KOOS in patients with other knee complaints including osteoarthritis is therefore advised.

## Conclusion

In conclusion, the present study demonstrated the Polish version of the KOOS being a valid, reliable and responsive outcome measure in young patients having ACLR. The KOOS can be applied as a patient-reported and disease-specific instrument in future studies including ACL injured subjects in Poland.

## Abbreviations

PRO: Patient-reported outcome; KOOS: Knee injury and Osteoarthritis Outcome Score; ACLR: Anterior cruciate ligament reconstruction; ADL: Activities of daily living; QOL: Quality of life; SF-36: Short Form 36; PF: Physical functioning; RP: Role-physical; BP: Bodily pain; GH: General health; VT: Vitality; SF: Social functioning; RE: Role-emotional; MH: Mental health; ICC: Intraclass correlation coefficient; CI: Confidence interval; SEM: Standard error of measurement; MDC: Minimal detection change; MIC: Minimal important change; GPE: Global perceived effect; ES: Effect size; SRM: Standardized response mean; OA: Osteoarthritis.

## Competing interests

The authors declare no conflict of interest related to this manuscript.

## Authors’ contributions

PTP and EMR were responsible for conception and design of the study. DW performed the surgeries. RK drafted the questionnaires. All authors participated in acquisition and interpretation of data. PTP performed statistical analyses. The article was drafted by PTP and was revised critically for important intellectual content by EMR. All authors have approved the final version for submission.
